# Serial measurement of Wessex Head Injury Matrix in the diagnosis of patients in vegetative and minimally conscious states: a cohort analysis

**DOI:** 10.1136/bmjopen-2014-006051

**Published:** 2015-04-21

**Authors:** Lynne Turner-Stokes, Paul Bassett, Hilary Rose, Stephen Ashford, Aung Thu

**Affiliations:** 1 Department of Palliative Care Policy and Rehabilitation, King's College London, School of Medicine, London, UK; 2 Regional Rehabilitation Unit, Northwick Park Hospital, London, UK; 3 Statsconsultancy Ltd, London, UK

**Keywords:** Wessex Head Injury Matrix, Prolonged Disorders of consciousness, Vegetative State, Minimally Conscious State, Brain Injuries, Patient Outcome Assessment

## Abstract

**Objective:**

To evaluate serial application of the Wessex Head Injury Matrix (WHIM) in diagnosis of prolonged disorders of consciousness (PDOC). Specifically, to determine whether the trajectory of change predicts outcome status, and whether the current hierarchical order of WHIM items is correct for this context.

**Design:**

Analysis of prospectively gathered clinical cohort data.

**Setting:**

Consecutive admissions to a tertiary in-patient neurorehabilitation service for evaluation of PDOC in real-life clinical practice, over a 10-year period (2004–2014).

**Participants:**

Patients (n=65) presenting in sudden-onset vegetative (VS) or minimally conscious states (MCS). Mean age 38.4 (sd14.1) years; male:female ratio 66%:33%. Aetiology of brain injury: 40(62%) traumatic; 12(19%) vascular; 11(17%) hypoxic; 3(3%) other.

**Primary outcome measure:**

WHIM alongside detailed clinical evaluation.

**Methods:**

The WHIM was administered serially by the multidisciplinary team throughout an in-patient evaluation programme (mean length 74 (sd42) days). Patients were divided into four groups, according to PDOC status on discharge (VS, MCS-Minus, MCS-Plus or Emerged).

**Results:**

WHIM hierarchical scores (Most Advanced Behaviour (MAB)) correlated with PDOC status at discharge (Pearson r=0.49, p<0.001). In the original order, the MAB distinguished the ‘VS’, ‘MCS’ and ‘Emerged’ categories (analysis of variance (ANOVA) post hoc p<0.001), but not the subgroups of MCS-Minus and MCS-Plus. In stepwise regression analysis, MAB-Ex (excluding two items) accounted for 68% of the variance in PDOC status at discharge. On multilevel statistical modelling, trajectory of change in MAB separated the four PDOC groups, both at individual and at group level (p<0.001). After reordering of items, the new-order MAB accounted for more (73%) of the variance in PDOC status, and also distinguished significantly between MCS-Minus and MCS-Plus groups at discharge (p<0.002).

**Conclusions:**

The WHIM is a useful diagnostic tool in PDOC, and trajectory of change is an important predictor of outcome. The proposed new hierarchical order requires further evaluation in future multicentre analyses.

**Strengths and limitations of this study**

- It is the largest cohort analysis of serial Wessex Head Injury Matrix (WHIM) data in the published literature so far.
- It represents use of the WHIM in the context of real-life clinical practice, over a substantial period of time (10 years).
- It has practical clinical application—the proposed new item order resonates with clinical experience and was shown to perform better in the diagnosis of different levels of prolonged disorders of consciousness (PDOC).
- It is a single-centre study—the generalisability of our findings requires further evaluation in multicentre studies.
- It does not include long-term follow-up, so the eventual outcomes for this sample are unknown.

## Introduction

Following sudden-onset severe brain injury, many patients progress through stages of vegetative (VS) and minimally conscious state (MCS) as they emerge into a state of full awareness. For some patients this can be a relatively quick transition, occurring over days or a few weeks. Others remain in prolonged disorders of consciousness (PDOC) for at least 4 weeks postinjury and progress more slowly over months or years. A few will plateau and remain in VS or MCS for the rest of their lives.^1^

The Aspen Group has defined operational criteria for the diagnosis of VS^2^ and MCS.^3^ VS is characterised by responses that are only ‘spontaneous or reflexive’, while MCS patients exhibit some inconsistent but reproducible ‘localising or discriminatory’ responses. MCS is a broad category encompassing the range from VS to emergence into consciousness. In recent years, the research group from Liege has proposed separation of MCS into two subgroups:^4^ ^5^ MCS-Minus (MCS−) patients show only lower level non-reflexive function, while MCS-Plus (MCS+) patients demonstrate more complex or purposeful behaviours, such as command following or intelligible verbalisation. Although the prognostic significance of these terms has not yet been fully explored, they are potentially useful at a clinical level for describing differential levels of interaction in MCS patients.

Accurate diagnosis is essential for clinical decision-making, treatment planning, prognostication—and sometimes also for legal purposes.^1^ At the current time, there is no single laboratory or clinical investigation that will confirm the diagnosis of VS or MCS. The diagnosis is made primarily on the basis of careful clinical evaluation by appropriately trained professionals.

Diagnosis rests on clinical observation of patient behaviours that may suggest some degree of awareness of themselves and/or their environment, but is challenging in this context for a number of reasons:

- Profound motor and sensory deficits, or indeed aphasia,^6^ may mask the behaviours that demonstrate awareness;^7^
- Responses are typically delayed and inconsistent in PDOC;^8^ ^9^
- Patients are sometimes assessed at too early a stage in their recovery, based on an insufficient period of observation and in the absence of a structured approach to evaluation.^10^

Misdiagnosis therefore remains a significant problem in PDOC,^8^ ^11^ ^12^ and may be the result of either diagnostic error or change in the patient's condition over time. Accordingly, the recent UK National Clinical Guidelines recommend the use of formal validated structured assessment tools as part of the detailed clinical assessment process to help to classify PDOC status appropriately.^1^ ^12^ Serial observation over time is also recommended to identify any trajectory towards more consistent or higher level responses. Because the emergence from coma or PDOC generally occurs through a gradual (and often variable) process of recovery, individual trend analysis may conceivably provide the best indication of outcome with respect to recovered awareness.^13^ ^14^

A large number of tools exist for the assessment and monitoring of PDOC. A recent review by the US taskforce^15^ identified 13 instruments, of which five were recommended for use in the evaluation of DOC with minor/moderate reservations. Of these, the JFK Coma Recovery Scale—Revised (CRS-R)^15^ ^16^ is most commonly used in the USA and parts of Europe, but the Wessex Head Injury Matrix (WHIM)^9^ ^17^ is the most commonly used instrument in the UK.^18^

The WHIM is a hierarchical scale, designed to provide a sequential framework against which to monitor changes in a individual's level responsiveness and interaction with their environment, as they progress from coma through to emergence from post-traumatic amnesia following traumatic brain injury.^9^ It may be applied by any member of the multidisciplinary team (MDT), and can even be used by family members as a framework for recording responses and interactions.^1^

Although it was not originally designed as a diagnostic tool for PDOC, the WHIM has proven to be a useful practical tool in the differential diagnosis of VS/MCS.

- Majerus *et al*^19^ validated a French version of the WHIM and broadly confirmed the hierarchical order of items, although the need for further development was highlighted.
- Schnakers *et al*^20^ used the operational definitions within the Aspen criteria^2^ ^3^ to separate WHIM items that may be used to distinguish VS from MCS.
- Wilson *et al*^17^ reported serial assessment with the WHIM as a tool to detect subtle changes in cognitive and communicative function over time.

As yet, however, there has been no formal attempt to map the WHIM items onto the criteria for different diagnostic subgroups in PDOC, based on a large series of observational data, nor to evaluate the trajectory of change as a predictor of outcome.

### Study objectives

In this study, we describe the serial application of the WHIM in a consecutive series of patients in PDOC states to address the following questions:

- Can the WHIM be used as an adjunct diagnostic tool for PDOC?
  - Which WHIM behaviours are compatible with the different diagnostic subgroups of VS and MCS?
  - Can it be used to distinguish between different PDOC states, including MCS− and MCS+?
- What are the patterns of change in WHIM scores recorded serially over time?
- Does either the baseline score or trajectory of change help to predict the individuals who eventually emerge into consciousness?
- Are the WHIM items in the correct hierarchical order—and, if not, what order would be more appropriate?

## Methods

### Design, setting and participants

A cohort analysis of prospectively collected serial WHIM data in consecutive patients admitted to a tertiary specialist neurorehabilitation unit in London, UK, for evaluation of PDOC during a 10-year period between 2004 and 2014. The programme consists of detailed clinical evaluation by a highly skilled MDT of therapists, doctors and nurses trained in the assessment and management of patients in PDOC. Care is first optimised through:

- A 24 h programme of postural management with changes of position as appropriate (lying, sitting, standing);
- Review of medications to minimise any sedative effects;
- A controlled programme of social stimulation with planned rest periods, tailored to the needs of the individual.

Routine evaluation includes detailed clinical assessment by the various members of the MDT, and serial structured assessment using the WHIM, and latterly the Sensory Modality Assessment and Rehabilitation Technique (SMART)^21^ and CRS-R.

As this was an observational study of real life clinical practice, the assessments were not conducted at any fixed time point. The timing for admission to and discharge from the programme depended on when the patient was referred, the waiting list and any external constraints such as time-limited funding.

Patients were included if they were admitted in either VS or MCS as a result of sudden-onset acquired brain injury (any aetiology), and had at least three WHIM assessments. All WHIM assessments recorded during the evaluation period were included for each patient. The median period of observation for each WHIM rating was 30 min (IQR 20–40) and the median number of assessments per patient was 26 (IQR 13–36, range 4–70). (NB The patients who had only a very small number of assessments either died (n=2) or (more commonly) emerged early in the programme so that WHIM recordings were discontinued).

### Diagnostic definitions

The primary diagnostic categorisation of patients as VS/MCS was by clinical diagnosis, based on holistic multidisciplinary clinical evaluation in relation to the following criteria:

- VS was diagnosed in accordance with the criteria laid down by the Royal College of Physicians.^1^ ^22^
- MCS was diagnosed in accordance with the Aspen Neurobehavioral Conference Workgroup^3^ based on the presence of inconsistent but reproducible responses at a localising or discriminating level—the hallmark of MCS being inconsistency.^1^
- Emergence from MCS was also defined according to the Aspen criteria^3^—namely reliable and consistent demonstration of one or both of the following:
  *Functional interactive communication*—which may occur through verbalisation, writing, yes/no signals or use of augmentative communication devices to answer 6/6 questions correctly on two consecutive occasions.
  *Functional use of objects*—which requires the demonstration of consistent behavioural evidence of discrimination between at least two different objects consecutively.

Operational criteria for consistency were applied according to the slightly extended set published in the national guidelines.^1^

In this service, the categorisation of PDOC status is assigned at discharge from the programme by consensus of the experienced MD clinical team, based on the findings from the full clinical evaluation, including any structured assessments that were undertaken.

Unlike the definitions of VS and MCS, detailed operational definitions for MCS+ and MCS− are not yet fully defined. Nevertheless, we believe the separation may be helpful as MCS is otherwise an extremely broad category reflecting any state between VS and emergence into consciousness. Since 2012, patients in MCS are routinely subcategorised into MCS+ and MCS−. For the purpose of this evaluation, subcategorisation was applied retrospectively for admissions prior to 2012. This was achieved through examination of the clinical records (including structured assessments).

- MCS− was assigned where only lower level non-reflexive function was recorded, for example:
  Localisation to noxious stimuli,*
  Pursuit eye movements,*
  Auditory tracking (ie, following the source of a sound),Ψ
  Affective behaviours (eg, smiling or crying in relation to environmental stimuli).*
- MCS+ was assigned where there was evidence of more complex or purposeful, but still inconsistent behaviours such as:
  Command following,*
  Intelligible verbalisation,*
  Reproducible non-verbal communication (eg, through gesture, blinks etc),*
  Occasional functional use of objects.Ψ

The behaviours marked with ‘*’ are the operational criteria in the original descriptions of MCS−4 5 which largely rely on item definitions in the CRS-R—a tool which is not commonly used in the UK. The items marked ‘Ψ’ are additional behaviours that are frequently observed in our wider clinical evaluation of MCS patients. They deserve brief explanation:

- In clinical terms, ‘auditory tracking’ is a step above the crude ‘localisation of sound’ that may be spontaneous or reflexive and is therefore identified as compatible with VS in the CRS-R scale. Auditory tracking is a potentially important localising response in patients who have impaired/absent vision, and frequently occurs in patients whose responses fall short of command following or functional communication. Hence, we placed patients with this criterion in the MCS− category.
- Similarly the occasional functional use of objects, falling short of the consistency required to fulfil criteria for emergence, was considered to fit best within the category of MCS+.

### Measures

#### Wessex Head Injury Matrix

The WHIM is a 62-item hierarchical scale, which provides a sequential framework of tightly defined categories of observation covering an individual's level responsiveness and interaction with their environment.^23^ According to the instruction manual,^23^ the team administers the scale by observing patients’ behaviours in the context of an interactive session and recording these as they are categorised in the WHIM scale. Periods of observation may vary from a few minutes to several hours, but in this evaluation was typically 20–40 min. Recorded behaviours are ticked off on the checklist if they meet the operational definitions. Those not observed are marked ‘x’. Hierarchical summary scores recorded for the session are (A) the ‘Most Advanced Behaviour’ (MAB) observed and (B) the ‘Total Number of different Behaviour items’ (TNB) observed (ie, the range of behaviours).

On this unit, all therapy staff are routinely trained in the use of the WHIM through regular in-house training sessions. Permanent nursing staff can also access this training. WHIM scores may be recorded by any member of the MDT who has undergone the requisite training. There is no set recording frequency—instead we aim to capture ratings at different times in the day, recording interaction with a range of different professionals. WHIM rating is discontinued when either (A) the patient is discharged from the unit, (B) assessment is considered complete or (C) the patient emerges from PDOC.

### Analysis

Data are systematically collated from the patient clinical records in Microsoft Excel at the time of discharge. After cross-checking, cleaning and validation against the unit records to ensure that data entry was as accurate and complete as possible, the data set was transferred to SSPS V.21 for analysis.

1. Because items 26 (‘Frowning and grimacing’) and 43 (‘Smiles for any reason’) are noted by both Schnakers *et al*^20^ and the RCP guidelines to be potentially out of order in the hierarchy,^1^ we also recorded the ‘MAB excluding items 26 and 43’ (MAB-Ex) for each WHIM rating, in addition to the MAB and TNB.
2. Although the WHIM generates ordinal data, the data set was large and inspection of histograms showed that distribution of the MAB, MAB-Ex and TNB were all within acceptable limits of normality, so parametric statistics were used throughout.
3. Demographics were summarised with descriptive statistics (%, mean, SD and range). Patients were divided into four groups according to their PDOC status (VS, MCS−, MCS+ or Emerged) on admission and discharge. One-way analysis of variances (ANOVAs; for continuous variables) and χ^2^ tests (for categorical variables) were used to test for group differences in demographic characteristics and baseline WHIM scores on admission.
4. To determine whether the WHIM can be used to distinguish the different categories of PDOC (VS, MCS−, MCS+ or Emerged), one-way ANOVAs with post hoc Bonferroni correction were used to examine group differences on admission to, and discharge from, the programme.
5. To examine the patterns in the trajectory of change in WHIM scores, individual serial ratings were first plotted for each individual over time. Serial data were then summarised for each individual by recording the average WHIM hierarchical scores within each month of the programme (up to 6 months).
6. To determine whether baseline parameters could help to predict the individuals who eventually emerge into consciousness, Pearson correlations were used to identify baseline measures that were significantly associated with PDOC status on discharge. Significant factors were then entered into stepwise multiple regression model to determine the best predictors both at baseline and at discharge.
7. In a longitudinal analysis of month-by-month data, multilevel linear regression modelling was used to compare the trajectories of change within each group for MAB and TNB. Two-level models were used with individual measurements nested within patients. The terms included in the model were time (as a continuous variable), patient group and the interaction between time and group. A significant interaction would suggest that the change in values over time varied between groups. A random patient slope for time (allowing the slope for each patient to vary over time) was also included when this gave a significant improvement to the model, which it did in the case of TNB, but not MAB.
8. To determine whether WHIM items are in the correct hierarchical order, we conducted an item-level pooled analysis of all 1668 WHIM ratings. The frequency of observation for each item was collated and mapped against the RCP criteria and the item groupings proposed by Schnakers *et al*.^20^ Item frequency within each of the PDOC diagnostic groups at discharge (VS, MCS−, MCS+) was then used to examine and revise the hierarchical order of the items.
9. Reanalysis was then conducted as per 4 and 6 above, using the reordered hierarchical scores.

## Results

Of 68 patients admitted for evaluation of PDOC during this period, 2 had only one WHIM score and data were missing for another, leaving a total of 65 patients for analysis. The demographics of the sample are shown in table 1. The mean age was 38 (SD 14.1) years, and two-thirds of the population was male. The aetiology of brain injury was traumatic in 62% and non-traumatic in 38%. The mean time from onset to admission was 16.2 weeks (range 2–120), but evaluation did not start until at least 4 weeks after injury. The mean period of the PDOC observation programme was 74 days (SD 42, range 6–209) and the mean time from injury to end of the assessment period was 6.2 months (SD 3.8, range 2.0–30.2). One-way analysis of variance tests revealed no significant interaction between PDOC status on admission and age, gender, time since onset or length of the observation period.

**Table 1:** Demographic characteristics of the sample on admission

Demographic		Study sample (n=65)			
		Mean (SD)		Range	
Age (years)		38.4 (14.1)		16–71	
Time since onset of injury (weeks)		16.2 (15.6)*		2–120	
Length of period observed (days)		74 (42)		6–209	
Total length of stay in rehabilitation unit (days)		127 (70)		22–393	
		N (%)			
Gender (male:female ratio)		43:22 (66%:33%)			
Aetiology					
Trauma		40 (61.5%)			
Vascular		12 (18.5%)			
Hypoxia		11 (16.9%)			
Other†		3 (3.0%)			

PDOC diagnosis on admission		PDOC diagnosis by discharge			
		VS	MCS−	MCS+	Emerged

VS	30 (46.2%)	12	10	3	5
MCS−	19 (29.2%)	–	2	5	12
MCS+	16 (24.6%)	–	–	7	9
	Total	12 (18.5%)	12 (18.5%)	15 (23.1%)	26 (40.0%)

*One extreme outlier with a time since onset of 6 years was excluded.

†Other aetiologies included hypoglycaemia (n=1) and encephalitis (n=1).

MCS, minimally conscious state; MCS−, MCS-Minus; MCS+, MCS-Plus; PDOC, prolonged disorders of consciousness; VS, vegetative state.

Based on the holistic clinical evaluation, 30 (46%) patients were categorised as in VS on admission, 19 (29%) as MCS− and 16 (25%) as MCS+. Table 1 also indicates the number in each group who progressed to a higher level by discharge. In all 40% had emerged into consciousness. In this sample there was no significant difference in outcome (emergence from PDOC) between patients with traumatic/non-traumatic aetiology.

### Group differences for PDOC status at discharge

Table 2 shows the mean WHIM ratings (MAB, MAB-Ex and TNB) at admission and discharge from the programme, grouped according to PDOC status at discharge. At admission, significant group differences were seen on post hoc tests between MCS− and MCS+ (p≤0.01), but not between the VS and MCS− groups, nor between MCS+ and those who had emerged by discharge. By discharge, group differences had widened. Significant differences in MAB and MAB-Ex were seen between each of the three broad groups—VS, MCS and Emerged (p<0.001)—but not now between MCS− and MCS+.

**Table 2:** Mean WHIM hierarchical scores (original order) on admission and discharge and group comparison using one-way ANOVA

Grouped according to PDOC status at discharge	VS (n=12)		MCS− (n=12)		MCS+ (n=15)		Emerged (=26)		One-way ANOVA	Post hoc tests with Bonferroni correction			
	Mean (SD)	Range	Mean (SD)	Range	Mean (SD)	Range	Mean (SD)	Range	p Value	Pairs	Mean difference	95% CI	p Value*
Admission scores													
MAB	11.3 (9.7)	2–26	10.8 (8.3)	3–26	24.4 (10.0)	8–43	25.3 (12.8)	1–50	<0.001	VS vs MCS−	0.6	−11.6 to 12.8	1.0
										MCS− vs MCS+	−13.6	−25.2 to −2.1	**0**.**01**
										MCS+ vs Emerged	−0.91	−8.8 to 10.6	1.0
MAB-Ex	5.7 (4.3)	1–14	8.5 (4.7)	3–15	24.1 (10.0)	8–43	23.5 (13.7)	1–50	<0.001	VS vs MCS−	−2.8	−14.3 to 8.8	1.0
										MCS− vs MCS+	−15.6	−26.5 to −4.6	**0**.**002**
										MCS+ vs Emerged	−0.53	−8.6 to 9.7	1.0
TNB	4.2 (2.6)	1–11	5.0 (2.1)	2–8	13.7 (11.6)	4–52	12.7 (6.4)	1–24	<0.001	VS vs MCS−	−0.8	−8.7 to 7.0	1.0
										MCS− vs MCS+	−8.7	−16.1 to −1.2	**0**.**01**
										MCS+ vs Emerged	−1.0	−7.2 to 5.27	1.0
Discharge scores													
MAB	8.2 (9.8)	2–26	26.0 (6.8)	15–36	35.2 (10.3)	17–56	49.2 (12.2)	5–61	<0.001	VS vs MCS−	−17.8	−29.6 to −6.1	**0**.**001**
										MCS− vs MCS+	−9.2	−20.4 to 2.0	0.20
										MCS+ vs Emerged	−14.0	−23.4 to −4.7	**0**.**001**
MAB-Ex	4.6 (5.2)	2–21	26.0 (6.8)	15–36	35.1 (10.2)	17–56	48.3 (13.2)	5–61	<0.001	VS vs MCS−	−21.4	−33.1 to −9.8	**<0**.**001**
										MCS− vs MCS+	−9.0	−20.1 to 2.1	0.18
										MCS+ vs Emerged	−13.2	−32.3 to 12.3	**<0**.**001**
TNB	3.3 (1.6)	1–7	9.2 (3.8)	4–17	16.8 (5.7)	8–26	28.5 (9.7)	3–47	<0.001	VS vs MCS−	−5.8	−13.7 to −2.0	0.3
										MCS− vs MCS+	−7.6	−15.2 to −0.1	**0**.**05**
										MCS+ vs Emerged	−11.7	−18.0 to −5.3	**<0**.**001**

*Significant p values after Bonferroni correction are highlighted in bold.

ANOVA, analysis of variance; MAB, Most Advanced Behavior; MAB-Ex, Most Advanced Behaviour excluding items 26 and 43; MCS, minimally conscious state; MCS−, MCS-Minus; MCS+, MCS-Plus; PDOC, prolonged disorders of consciousness; TNB, Total Number of Behaviours; VS, vegetative state; WHIM, Wessex Head Injury Matrix.

In order to determine the extent to which outcome (PDOC status at discharge) could be predicted from baseline data, we examined the correlation with age, time since onset, length of observation period and admission WHIM ratings. Only the WHIM ratings (MAB, MAB-Ex and TNB) were correlated significantly (r=0.49, 0.58 and 0.46, respectively (all p <0.001)). When these were entered stepwise as independent variables in a multiple regression model, only the MAB-Ex was included the model, accounting for 32% of the variance. When WHIM ratings at discharge were entered as variables, MAB-Ex was entered first accounting for 68% of the variance, and TNB second accounting for a further 5% (73% together). MAB was excluded from the model. This suggests that at least some of the items are out of order.

### Serial WHIM scores—trajectories of change

Serial rating of the WHIM in individual patients over the course of their assessment period produced a number of distinctive trajectory patterns of which four examples are shown in online supplementary file 1.

**Figure 1:**
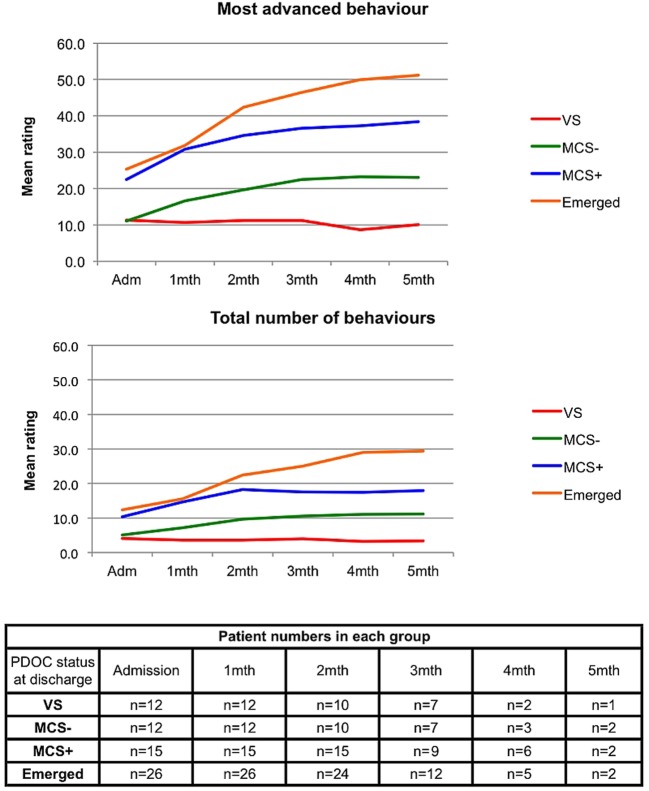
Group trajectories of change in serial WHIM ratings categorised by PDOC status at discharge. Figure shows the progression of the mean Most Advanced Behaviour and Total Number of Behaviours on serial ratings over time, grouped by patient status at discharge. Patient numbers for whom data were recorded each month are shown in the table. Where patients had either been discharged or WHIM recording had ceased, the final WHIM scores were carried forward. MCS, minimally conscious state; MCS−, MCS-Minus; MCS+, MCS-Plus; PDOC, prolonged disorders of consciousness; VS, vegetative state; WHIM, Wessex Head Injury Matrix.

Figure 1 summarises the group trajectories of change in serial WHIM ratings (mean monthly MAB and TNB), categorised by PDOC status at discharge. Only a small minority of patients (n=16) were still undergoing testing by the fifth month, a diagnosis having been reached for the majority by month 3–4. For the purposes of illustration, the final ratings were carried forward in the graph. Multilevel linear regression modelling was used to compare the trajectories of change for MAB and TNB. For the statistical modelling, no missing data were imputed, but the analysis was restricted to the baseline and four subsequent months, given the small numbers of patients remaining in the programme thereafter.

There were highly significant group-by-time interactions for both outcomes, indicating that the rate of change over time varied significantly between the four groups (see table 3). As expected, for both outcomes, the VS group had the lowest change and this group did not change significantly over time for either outcome (the CIs for the rate of change crossing zero). Rates of change for both parameters increased progressively towards the highest level of PDOC status (ie, Emerged), confirming that the trajectory of change is an important indicator of eventual outcome with respect to recovered awareness.

**Table 3:** The results of multilevel linear regression to examine rate of change over time for the four groups of patients at discharge

Outcome	Group	Rate change/month estimate (95% CI)	Difference mean (95% CI)	Interaction p Value
MAB	VS	−0.9 (−2.8 to 1.0)	0	<0.001
	MCS−	4.8 (2.9 to 6.6)	5.7 (3.0 to 8.3)	
	MCS+	3.4 (2.0 to 4.9)	4.3 (1.9 to 6.7)	
	Emerged	8.4 (7.1 to 9.7)	9.3 (7.0 to 11.6)	
TNB	VS	−0.2 (−2.3 to 1.8)	0	<0.001
	MCS−	1.7 (−0.3 to 3.8)	1.9 (−1.0 to 4.8)	
	MCS+	2.8 (1.1 to 4.5)	3.0 (0.4 to 5.7)	
	Emerged	6.0 (4.6 to 7.4)	6.2 (3.8 to 8.7)	

MAB, Most Advanced Behavior; MCS, minimally conscious state; MCS−, MCS-Minus; MCS+, MCS-Plus; TNB, Total Number of Behaviours; VS, vegetative state.

### Item by item analysis—evaluation of the WHIM hierarchical order

Across the whole sample, a total of 1668 individual WHIM assessments were recorded—336 in patients who were discharged in a vegetative state; 792 in patients discharged in a minimally conscious state (309 in MCS− and 483 in MCS+); and 540 in patients who emerged.

Online supplementary files 2 and 3, respectively, show the per cent frequency of WHIM behaviours observed (n=1668 ratings) and the percentage frequency of WHIM items in patients who were in VS throughout their admission, compared with those who were in MCS or had emerged at discharge. As expected, those who emerged showed higher frequency of behaviours across the range of the WHIM than those in MCS, but the order of frequency was broadly similar.

#### Vegetative state

Online supplementary file 2 also shows the frequency of behaviours observed in those patients who remained in VS (n=366 ratings) listed alongside the behaviours that would be theoretically compatible according to the RCP criteria,^22^ and the operational criteria used by Schnakers.^20^

In this series, only nine items (1–5, 7, 8, 14 and 26) were recorded on >3% (12 or more) occasions in patients who remained in VS. A further six items (6, 9, 11, 13, 15 and 24) were recorded on 3–12 occasions. Two items that are compatible with VS according to both Schnakers^20^ and RCP criteria,^22^ were seen either rarely (No 21 ‘Crying’ n=2) or not at all (No 43 ‘Smiles for any Reason’ n=0) in this series. Other items that could theoretically be compatible (according to Schnakers: No 19 ‘Speaks in whispered tones’, No 20 ‘Vocalises to express moods/needs’, No 30 ‘Laughs’; and according to the RCP criteria No 10 ‘Expletive utterance’) were not seen in VS patients in this sample.

As expected, Item 26 was recorded on as many as 11% occasions confirming that it is out of order in the hierarchy. The recording on six occasions of item 15 (Performs physical movement on verbal request) was at first sight surprising. However, it occurred only 1–2 times in four individuals against a background of spontaneous movement, which is sufficiently uncommon to be considered a chance finding.

#### Minimally conscious states

Ten further items (12, 15, 16–18, 22, 23, 28, 31, 33) were seen in >3% of patients with MCS−. In addition to items 19, 20, 30 and 43 aforementioned, nine items (25, 27, 29, 32, 34–6, 38, 41) were seen in >3% MCS+. A further 17 items were seen occasionally in patients with MCS+ but, in this sample, items 55 and 58–62 were seen only in patients who emerged.

These findings confirm that while the original WHIM hierarchical order was generally associated with the level of responsiveness, some items were out of order, so limiting its use as a diagnostic tool.

### Proposed reordering of items and preliminary testing

**Figure 2:**
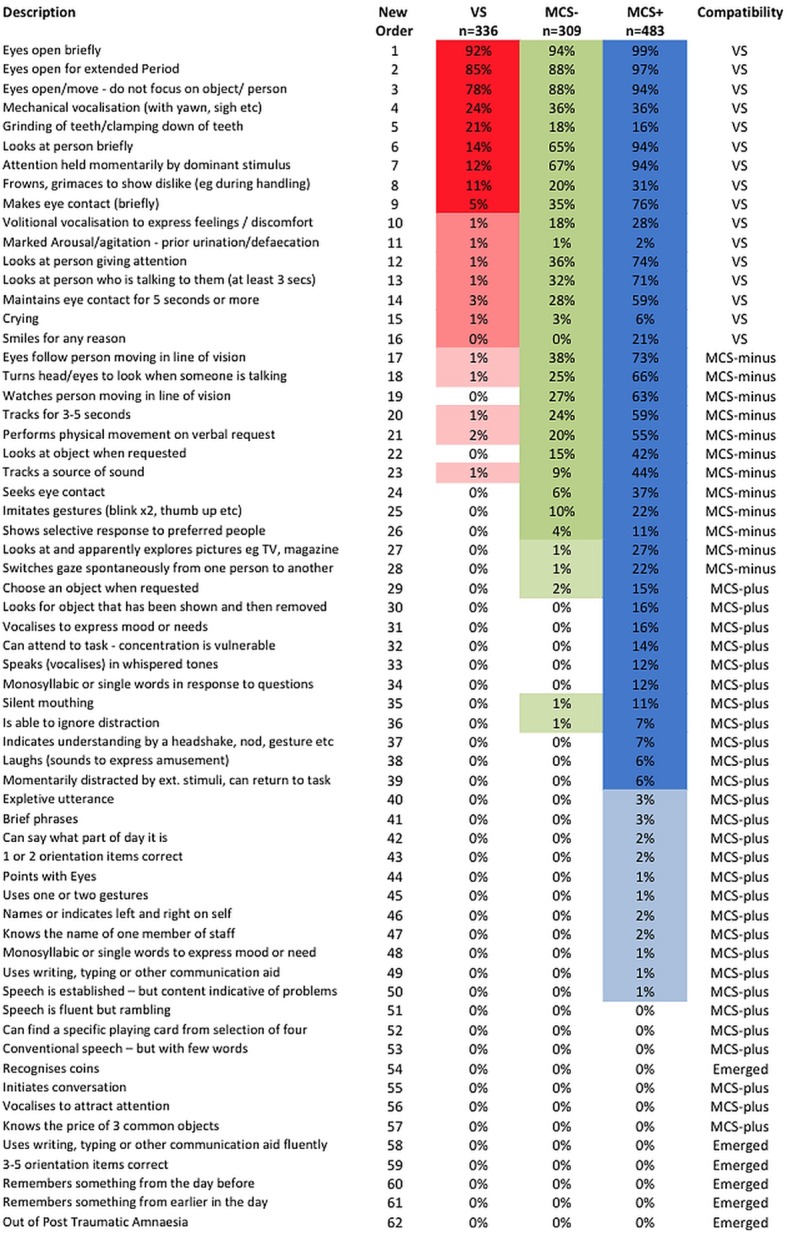
Proposed reordering of WHIM items. Figure shows the proposed new order for WHIM items listing the frequency in which they were observed in patients remaining in VS, MCS− and MCS+ at discharge. MCS, minimally conscious state; MCS−, MCS-Minus; MCS+, MCS-Plus; VS, vegetative state; WHIM, Wessex Head Injury Matrix.

**Figure 3:**
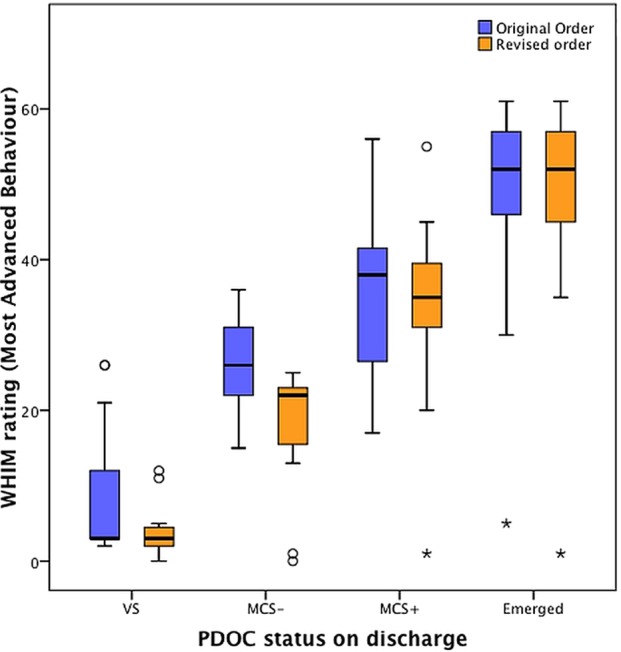
Distribution of Wessex Head Injury Matrix (WHIM) ratings at discharge categorised by prolonged disorders of consciousness (PDOC) status: comparison of the original and revised order. Figure shows the Box and Whisker plots for distribution of the ‘Most Advanced Behaviour’ recorded at discharge grouped by PDOC status at discharge. Although there is still some overlap at the end of range, the revised order provides better separation of the four groups.

Figure 2 sets out a proposed new order of items, listing those that were observed in patients remaining in VS, MCS− and MCS+ at discharge. Items were ordered (by a combination of the frequency of their occurrence and theoretical compatibility^1^ ^20^) first by patients remaining in VS, then by those remaining in MCS− and finally by those remaining in MCS+. Having derived the new order, we re-ran the analysis shown in table 2, using the ‘New-order MAB’. Significant differences were now seen between all four groups at discharge (see table 4, illustrated in figure 3). When this ‘new-order MAB’ on discharge was entered into the stepwise regression model alongside the original order parameters (MAB and TNB), the ‘new-order MAB’ was returned first accounting for 73% of the variance in PDOC status, with TNB accounting for a further 3%, but the original order MAB was excluded.

**Table 4:** Mean WHIM hierarchical scores (new order) on admission and discharge and group comparison using one-way ANOVA

Grouped according to PDOC status at discharge	VS (n=12)		MCS− (n=12)		MCS+ (n=15)		Emerged (n=26)		One-way ANOVA	Post hoc tests with Bonferroni correction			
	Mean (SD)	Range	Mean (SD)	Range	Mean (SD)	Range	Mean (SD)	Range	p Value	Pairs	Mean difference	95% CI	p Value*
Admission scores													
MAB (new-order)	7.0 (6.9)	2–25	10.2 (6.7)	0–21	21.9 (11.3)	3–40	24.2 (13.5)	1–53	<0.001	VS vs MCS−	−3.1	−15.3 to 9.1	1.0
										MCS− vs MCS+	−11.8	−23.3 to −0.2	**0**.**04**
										MCS+ vs Emerged	2.3	−12.0 to 7.4	1.0
Discharge scores													
MAB (new-order)	4.0 (3.8)	0–12	17.9 (8.8)	0–25	33.8 (12.6)	1–55	49.3 (12.4)	1–61	<0.001	VS vs MCS−	−13.9	−25.9 to −1.9	**0**.**015**
										MCS− vs MCS+	−15.9	−27.3 to −4.5	**0**.**002**
										MCS+ vs Emerged	−15.5	−25.0 to −6.0	**<0**.**001**

*Significant p values after Bonferroni correction are highlighted in bold. ANOVA, analysis of variance; MAB, Most Advanced Behavior; MCS, minimally conscious state; MCS−, MCS-Minus; MCS+, MCS-Plus; PDOC, prolonged disorders of consciousness; VS, vegetative state; WHIM, Wessex Head Injury Matrix.

## Discussion

This cohort analysis set out to examine serial application of the WHIM as a diagnostic tool in PDOC. We found a general relationship between WHIM hierarchical scores and the level of responsiveness. Both the MAB and the TNB were significantly different in the three main diagnostic categories (VS, MCS and Emerged), but in the original order of the WHIM, the MAB did not distinguish between the subgroups of MCS− and MCS+.

We examined the patterns of change in WHIM scores recorded serially over time to determine whether the baseline data or trajectory of change would assist in identifying those individuals who emerge into consciousness by discharge. Baseline WHIM score accounted for only 33% of the variance in PDOC status at discharge. However, on longitudinal analysis, the rates of change for both MAB and TNB increased progressively across the four PDOC groups (from VS to Emerged) confirming that the trajectory of change is an important indicator of eventual outcome with respect to recovered awareness.

It is relevant to highlight the length of the evaluation window in comparison to some other settings. Giacino *et al*^10^ report a ‘nihilistic attitude’ to patients in PDOC and their exclusion from rehabilitation in some health cultures—also noting that, even if they are lucky enough to access rehabilitation, the standard 6-week programmes available in the USA are very often incompatible with the course of recovery in this group. By contrast, the UK provides relatively well for patients with PDOC. Our cohort had on average 4 months of intensive treatment in the acute setting, followed by 2–7 months of detailed evaluation under optimised conditions before reaching a clinical diagnosis. The total length of stay on the unit extended beyond the formal PDOC evaluation programme for many patients (mean 127 days, range 22–393), especially for those who emerged into consciousness. Moreover, in the UK, patients remaining in PDOC are generally entitled to 100% funded NHS Continuing Care (life-long if necessary), with ongoing assessment and review (see below).

Our pooled analysis of item frequency observed in all 1668 WHIM assessments, confirmed that the WHIM could potentially be used to categorise patients into the different PDOC subgroups, but that some reordering was required. Taking together the frequency of observation and the compatibility of items with different PDOC states as reported elsewhere^1^ ^20^ ^22^ we proposed a new hierarchical order for the WHIM. When reanalysed using this order, the ‘new-order MAB’ not only accounted for a larger proportion of the variance in PDOC status at discharge (73% compared with 68%), but it now distinguished significantly between MCS− and MCS+ groups (mean difference −15.9, 95% CI −27.3 to −4.5, p=0.002). Figure 3 illustrates the improved separation between the different diagnostic categories with this new order. While relatively modest at a statistical level, this improvement may potentially assist in the clinical diagnosis for at least a proportion of cases.

Our findings therefore concur with those of Majerus *et al*^19^ and Schnakers *et al*^20^ who broadly confirmed the hierarchical order of items and reported that WHIM items that may be used to distinguish the conditions of coma, VS and MCS. However, as acknowledged by Shiel *et al*^23^ in their original paper, the original order of items was not necessarily considered to be definitive. Similarly, the proposed new order presented here may not prove to be the final one, but our findings do at least demonstrate that the performance of the WHIM as a diagnostic tool can be improved by adjustment of the item order.

Although one previous analysis has recorded change in WHIM score from first to final assessment in 12 patients with PDOC,^17^ this paper provides the first longitudinal analysis to confirm the trajectory of serial WHIM assessments as an important indicator of outcome.

The authors recognise a number of limitations to this study:

1. This is the largest cohort analysis of serial WHIM data in the published literature so far. Nevertheless it is a single-centre study and the generalisability of our findings requires further evaluation in multicentre studies.
2. The data from this observational evaluation were collected in the course of real-life clinical practice, which confers both advantages and disadvantages. If a tool is to be used longitudinally over a period of months or even years to monitor the trajectory of change, it will inevitably be applied by many different people under changing conditions. On the positive side our findings are reflective of the WHIM as it is actually used in the clinical setting, documented over a decade by many staff members, during which its usefulness has stood the test of time. On the negative side, analysis has had to take account of changes in the way data are recorded in routine practice, which has become more sophisticated and rigorous over time. For example, other structured tools have gradually been introduced within in our clinical assessment process:
  - The SMART^21^ was introduced in 2007 after key team members became accredited SMART assessors. The SMART is now used to confirm a clinical diagnosis of VS, wherever this is suspected clinically, but is not routinely applied in all patients with PDOC in our service due to constraints on time and resources.
  - Routine use of the CRS-R^16^ and systematic recording of the Aspen criteria for emergence^3^ were introduced in our centre in 2012. Prior to that, ‘Emergence’ was diagnosed more informally from the clinical assessment of consistency in function and/or communication, and from other standardised measures, such as the UK Functional Assessment Measure^24^ which has been recorded for all patients in our service since 1996.

Therefore, the categorisation of PDOC status on admission and discharge was by the best methods available at the time. In the process of cleaning the data set, however, we reviewed the diagnosis of PDOC status in conjunction with the full clinical records to ensure that it was as accurate as possible. This included the subcategorisation of MCS into MCS+ and MCS− for patients admitted prior to 2012.

- The assignation of PDOC status discharge is based on the full clinical evaluation, including the WHIM and any other structured tools that were applied. We accept therefore a degree of circularity in the relationship between the WHIM and PDOC status at discharge. However, we would emphasise that in line with the UK guidelines, the clinical evaluation is conducted over very many hours using a wide range of techniques, of which WHIM rating forms only a relatively small part. Moreover, it was not our intention to propose the use of the WHIM as a stand-alone diagnostic tool, but simply to determine the extent to which it reflects the final diagnosis, and whether its performance in this respect could be improved by reordering.
- It should also be noted that the classification of PDOC at the end of the assessment programme is not necessarily a final condition. We do not know what the longer term outcomes for this sample were. The unit serves a large supra-regional catchment area and, until very recently, there has been no contract in place to support longer term evaluation of PDOC status after patients leave the in-patient evaluation programme. Some of the patients who were in MCS (or even VS) at the end of the programme may yet have emerged further on down the line, while some others will sadly have died.

The recently published National Clinical Guidelines for management of PDOC^1^ in the UK highlight the need for follow-up evaluation at regular intervals (6–12 monthly until the patient either emerges or a diagnosis of permanent VS or MCS is made), and also the systematic collection of longitudinal data to evaluate outcome, including application of structured assessment tools. It is anticipated, therefore, that systematic follow-up will become normal practice in the course of the next decade, and plans are underway for development of a national clinical database to support longitudinal data collection and future multicentre analysis. It is not suggested that use of the WHIM could replace the need for careful clinical assessment, but these findings provide support for its serial application as part of that evaluation.

### Directions for future research

Within this analysis we have concentrated on the order of items, rather than item redundancies. However, some items were rarely recorded suggesting that they may be redundant. In addition, the team highlighted others that are very similar, if not indistinguishable, at a clinical level. As well as reviewing the proposed new order, future analyses should explore the possibility of item reduction and also tightening the operational parameters for item rating within the WHIM manual.

The group that originally developed the WHIM is currently reviewing the item order and any possible item redundancies with a view to developing a second edition of the WHIM. This paper provides an independent analysis and a first step towards that further development. In addition, our large data set will provide a useful resource for future testing of any further proposals for revised item ordering.
